# Traumatic brain injury in patients with mechanical heart valves on lifelong anticoagulation: incidence, management, and outcomes of intracranial hemorrhage

**DOI:** 10.3389/fcvm.2026.1874285

**Published:** 2026-07-23

**Authors:** Jastina Feiereisen, Rasha E. Boulos, Can Keskinaslan, Yannik Kalbas, Hans-Christoph Pape, Tobias Gossler, Milan Milojevic, Igor Tudorache, Petar Risteski, Omer Dzemali, Hector Rodriguez Cetina Biefer

**Affiliations:** 1 Department of Cardiac Surgery, University Hospital Zurich, Zurich, Switzerland; 2 Department of Trauma, University Hospital Zurich, Zurich, Switzerland; 3 Institute of Anaesthesiology and Perioperative Medicine, University Hospital Zurich, University of Zurich, Zurich, Switzerland; 4 Department of Cardiac Surgery, City Hospital Zurich – Triemli, Zurich, Switzerland; 5 Center for Experimental and Translational Cardiology (CTEC), University of Zurich, Zurich, Switzerland; 6 State University of Tetova, Faculty of Medical Sciences, Tetova, North Macedonia

**Keywords:** anticoagulation, intracranial hemorrhage, mechanical heart valve, outcome, traumatic brain injury, vitamin K antagonist

## Abstract

**Objectives:**

Patients with mechanical heart valves require lifelong anticoagulation, exposing them to a substantially elevated risk of hemorrhagic complications following traumatic injury. Traumatic brain injury (TBI) in this population represents a particularly challenging clinical dilemma, as the coexistence of intracranial hemorrhage and the obligatory need for anticoagulation places competing physiologic imperatives in direct conflict. Despite the clinical relevance of this scenario, data on the incidence, management, and outcomes of TBI in anticoagulated mechanical valve patients remain scarce. This study aimed to characterize the incidence and severity of TBI and intracranial hemorrhage in this population, to evaluate the impact of hemorrhage on composite good outcome, and to compare outcomes between surgical and conservative management strategies.

**Methods:**

We conducted a retrospective observational cohort study of patients with mechanical heart valves who sustained at least one traumatic event at the University Hospital Zurich during 2012 and 2023. The primary outcome was a composite good outcome at one month, defined as absence of neurological sequelae, no new bleeding, and no progression of intracranial hemorrhage. Anticoagulation reversal strategies were recorded for all patients. Admission INR values were extracted from emergency laboratory records where available.

**Results:**

Among 60 patients with mechanical heart valves, 43 (71.7%) sustained at least one TBI, yielding 50 TBI events over the study period. The majority of TBIs were mild (90%), with a median patient age at first TBI of 73 years (interquartile range 56.5–79.8). Intracranial hemorrhage was present in 30 of 50 TBIs (60%). A composite good outcome was achieved in 18 of 20 TBIs (90%) without intracranial hemorrhage, compared with 20 of 30 TBIs (66.7%) with intracranial hemorrhage. Among hemorrhagic TBIs, good outcome was observed in 5 of 9 surgically managed cases (55.6%) and 15 of 21 conservatively managed cases (71.4%).

**Conclusions:**

Intracranial hemorrhage following TBI is common in anticoagulated mechanical valve patients and is associated with a lower rate of composite good outcome compared to TBI without hemorrhage (66.7% vs. 90.0%). Among haemorrhagic TBI events, composite good outcomes were numerically more frequent with conservative than with surgical management (71.4% vs. 55.6%); however, given the small number of surgical cases (*n* = 9), probable selection toward more severe haemorrhages in the surgical group, and the study's absence of statistical power to detect a clinically meaningful difference (*p* = 0.43), this comparison must be regarded as exploratory and hypothesis-generating only. No conclusions regarding the comparative effectiveness of conservative vs. surgical management can be drawn from this cohort. Admission INR data were available for 94% of TBI events (47 of 50). Median admission INR was 2.2 [IQR 1.6–3.1] overall; haemorrhagic TBI events had a significantly lower median admission INR than non-haemorrhagic events (1.8 [IQR 1.4–2.6] vs. 3.0 [IQR 1.9–3.3]; *p* = 0.025), suggesting that injury biomechanics rather than anticoagulation intensity alone determines haemorrhagic risk. These findings underscore the need for prospective multicentre data and standardised management protocols in this high-risk population.

## Introduction

Mechanical heart valve replacement remains the treatment of choice for a substantial proportion of patients with severe valvular heart disease, offering durable hemodynamic performance and long-term structural integrity. However, the thrombogenicity inherent to prosthetic valve surfaces mandates lifelong anticoagulation with vitamin K antagonists, a therapeutic necessity that exposes patients to a persistent and unavoidable hemorrhagic risk throughout their remaining lives (1–3). The balance between thromboembolic protection and bleeding risk constitutes one of the most consequential trade-offs in contemporary cardiovascular medicine, and it is a balance that becomes acutely destabilized in the setting of traumatic injury (4, 5).

Traumatic brain injury represents a leading cause of disability and death worldwide, with the majority of cases occurring in older individuals who are at disproportionately elevated risk of accidental falls (6). As the global population ages, the concurrent prevalence of anticoagulant therapy and fall-related injury has risen substantially, creating an expanding clinical challenge in emergency and surgical settings (6–8). In patients receiving therapeutic anticoagulation, even low-energy trauma carries a markedly elevated risk of intracranial hemorrhage, as the physiologic hemostatic response to vascular injury is fundamentally impaired (6, 7, 9). Hemorrhage may be immediate or delayed, and its clinical course is frequently unpredictable — secondary expansion remains a major determinant of neurological outcome, while delayed hemorrhage in initially CT-negative patients constitutes an additional and underappreciated source of morbidity (10, 11). Critically, however, the existing literature on anticoagulation-associated TBI is concentrated almost exclusively in elderly cohorts, driven by the epidemiology of fall-related injury in older adults and the high prevalence of anticoagulation for atrial fibrillation and venous thromboembolism in this age group (6, 12, 26). Patients with mechanical heart valves represent a fundamentally different population: valve replacement is frequently performed in younger and middle-aged patients, who then carry the dual burden of lifelong mandatory anticoagulation and decades of cumulative traumatic risk — a combination that is neither captured by elderly TBI cohorts nor addressable by the emerging literature on direct oral anticoagulants, from which mechanical valve patients are categorically excluded (1, 2, 13).

Among anticoagulated patients, those with mechanical heart valves therefore occupy a uniquely precarious position. Unlike patients anticoagulated for atrial fibrillation or venous thromboembolism, in whom therapy may be safely interrupted or reversed without immediate catastrophic risk, mechanical valve recipients face a competing and potentially life-threatening thromboembolic hazard with any interruption of anticoagulation, placing clinicians in a management dilemma for which robust evidence remains scarce (1, 2, 4, 14). The management of intracranial hemorrhage in this population demands simultaneous consideration of two otherwise conflicting imperatives — hemorrhage control and valve thrombosis prevention — for which no universally accepted protocol exists (2, 4, 15). The optimal timing of anticoagulation resumption after intracranial hemorrhage remains particularly contested, with emerging data suggesting that early resumption may be feasible but that heparin bridging during reinitiation carries an independent risk of hemorrhagic recurrence (16–18). Current evidence derives predominantly from case series and small retrospective cohorts focused on spontaneous intracerebral hemorrhage rather than traumatic intracranial hemorrhage, leaving the traumatic subgroup substantially undercharacterized across all age groups (1, 2, 19). Furthermore, while recent literature has increasingly examined the comparative safety of direct oral anticoagulants vs. vitamin K antagonists in the context of TBI, patients with mechanical heart valves — who remain mandatorily restricted to vitamin K antagonist therapy — represent a distinct and particularly vulnerable subgroup whose management cannot be extrapolated from the broader anticoagulated population (7, 12, 20).

The present study addresses these gaps directly. Using a retrospective cohort of patients with mechanical heart valves who sustained traumatic events over a defined study period, we sought to characterize the incidence and severity distribution of TBI and intracranial hemorrhage across a broad age spectrum, to evaluate the association between hemorrhagic TBI and composite good outcome at one month, and to compare outcomes between surgically and conservatively managed hemorrhagic injuries. Additionally, we examined the role of anticoagulation reversal strategies employed in the acute setting, with the goal of contributing to the evidence base needed to inform standardized management protocols for this mechanically anticoagulated, age-diverse, and clinically challenging population.

## Materials and methods

### Study design and setting

We conducted a single-center retrospective observational cohort study at the University Hospital Zurich, Zurich, Switzerland. Patients were identified through systematic cross-referencing of institutional surgical registry records, International Classification of Diseases (ICD) coding databases, and the institutional trauma registry from 2012 to 2023. This study was approved by the Cantonal Ethics Committee of Zurich (BASEC 2026-00876) and was conducted in accordance with the Declaration of Helsinki.

### Patient population

The sole inclusion criterion was hospitalization attributable to trauma combined with a documented history of mechanical heart valve replacement, regardless of valve position, patient age, or duration since implantation. Patients were included regardless of the number of traumatic events sustained during the study period. No exclusion criteria were applied. A total of 60 patients fulfilled the inclusion criterion and formed the study cohort.

### Data collection

Baseline patient characteristics, valve type and position, anticoagulation regimen, traumatic event details, clinical presentation, management strategy, and one-month outcomes were extracted from electronic medical records. For each patient, all traumatic events occurring within the study period were recorded individually, with particular attention to the radiological characteristics (subtype) of intracranial hemorrhage on admission computed tomography (CT) imaging. TBI severity was classified as mild, moderate, or severe according to Glasgow Coma Scale: mild GCS 13–15, moderate GCS 9–12, severe GCS 8 or less. Admission INR values at the time of TBI were extracted from emergency laboratory records in the institutional electronic health record for all TBI events. Repeat computed tomography of the head was performed on a case-by-case basis at the discretion of the treating team, guided by clinical indicators including neurological deterioration, new symptom onset, or clinical suspicion of haemorrhagic progression; no formal institutional protocol mandating routine repeat CT for all patients was in place during the study period, consistent with contemporary evidence supporting clinically indicated rather than universal protocolised repeat imaging (10, 11). Details of any anticoagulation reversal strategy administered during hospitalisation were recorded for all TBI events. Reversal agents documented included phytomenadione (Konakion), four-factor prothrombin complex concentrate (Beriplex), tranexamic acid, and fresh frozen plasma (FFP).

### Outcomes

The primary outcome was a composite good outcome at one month, defined as the simultaneous absence of new neurological sequelae, no new hemorrhagic event, and no radiological progression of intracranial hemorrhage on follow-up imaging. Outcome was assessed by a structured clinical review of inpatient records and one-month follow-up documentation. Given the retrospective design, neurological status was ascertained from available clinical documentation rather than by prospective application of a validated neurological scale; neither the modified Rankin Scale, Glasgow Outcome Scale, nor NIHSS was systematically documented for TBI management during the study period, precluding retrospective scale-based outcome classification. Complete one-month follow-up documentation was available for all 50 TBI events (100%); five events corresponded to patients who died before the 30-day endpoint and were classified as not achieving composite good outcome. Outcome assessors were not blinded to management strategy, as is inherent to the retrospective design. Secondary outcomes included the rate of composite good outcome stratified by the presence or absence of intracranial hemorrhage; the rate of composite good outcome by surgical vs. conservative management in hemorrhagic TBI; and the association between TBI severity and composite good outcome. Mortality prior to one-month follow-up and the need for reoperation were recorded as additional safety endpoints. Thromboembolic events occurring during TBI-related hospitalisations were recorded as safety outcomes.

### Statistical analysis

All statistical analyses were conducted using R software version 4.3.1. Continuous variables are presented as medians with interquartile ranges (IQR). Categorical variables are expressed as absolute and relative frequencies (%). Comparisons between groups for categorical outcomes were performed using Fisher's exact test. In computing the time elapsed between valve replacement and the first traumatic brain injury, procedure dates in the institutional cardiac surgical registry were available at the year level only; accordingly, the time-to-TBI interval was approximated as the difference between the calendar year of the TBI event and the calendar year of the index valve operation, introducing a maximum imprecision of plus or minus 1 year. This approximation applies exclusively to the descriptive temporal analysis; no statistical comparisons involving this variable were performed. Given the small number of moderate and severe TBI events (*n* = 4 and *n* = 1, respectively), any analysis of TBI severity and outcome is considered exploratory. A two-tailed *P*-value of less than 0.05 was considered statistically significant.

## Results

### Patient characteristics and valve distribution

**Figure 1:**
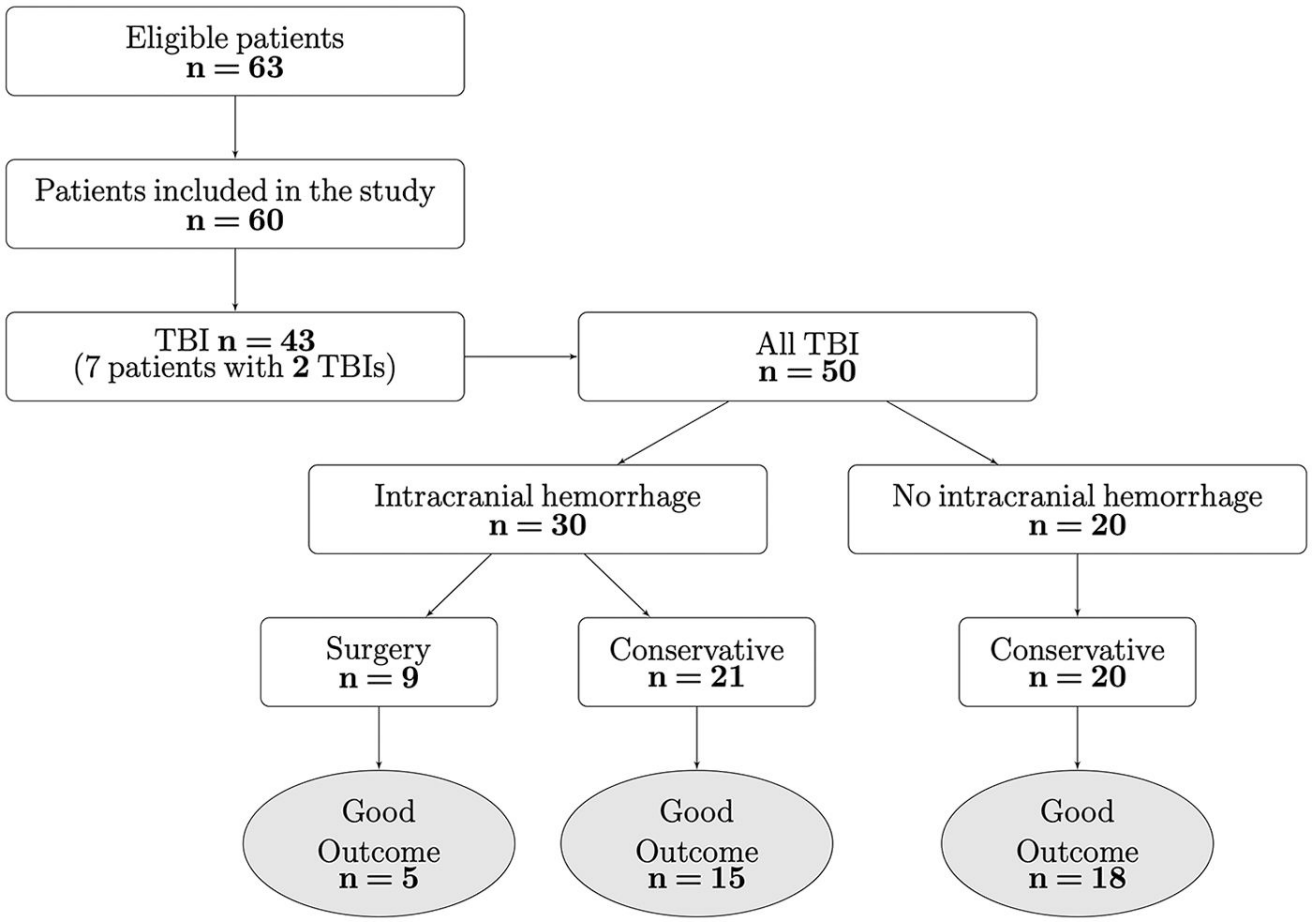
Flow diagram illustrating patient screening, inclusion, and distribution of traumatic brain injury events, intracranial hemorrhage, management strategy, and composite good outcome at one month. TBI, traumatic brain injury.

A total of 63 patients were screened for eligibility, of whom 3 didn't provide General Consent and were excluded; 60 patients fulfilled the inclusion criterion and were included in the final analysis (Figure 1). Among these, 41 patients (68.3%) had undergone aortic valve replacement, 14 (23.3%) had undergone mitral valve replacement, and 5 (8.3%) had undergone double valve replacement. The median age at the time of valve implantation was 58 years [IQR 46–64], and 40 patients (67%) were male (Table 1).

**Table 1:** Baseline patient characteristics.

Characteristic	Overall (*n* = 60)
Age at valve implantation, years, median (IQR)^a^	58 (46–64)
Sex, male, *n* (%)	40 (67%)
Mechanical valve position, *n* (%)	
Aortic	41 (68.3%)
Mitral	14 (23.3%)
Double	5 (8.3%)
Number of traumatic events, *n* (%)	
1	43 (72%)
2	12 (20%)
3	4 (6.7%)
≥ 4	1 (1.7%)
Number of TBI events per patient, *n* (%)	
0	17 (28%)
1	36 (60%)
2	7 (12%)
Comorbidities, *n* (%)	
Hypertension	36 (60.0%)
Diabetes mellitus	12 (20.0%)
Prior stroke or TIA	9 (15.0%)
Atrial fibrillation (any)	27 (45.0%)
Prior myocardial infarction	9 (15.0%)
Prior endocarditis	8 (13.3%)
Chronic kidney disease (eGFR <60 mL/min/1.73m2)^b^	19/49 (38.8%)
Antiplatelet co-therapy	0 (0%)
Prior intracranial haemorrhage	0 (0%)
Risk Scores	
CHA2DS2-VASc score, median (IQR)^c^	1 (1–3)
Modified HAS-BLED score, median (IQR)^c^	1 (0–2)

a Age at valve implantation: one value missing. IQR, interquartile range; TBI, traumatic brain injury.

b eGFR data available for 49 of 60 patients.

c CHA2DS2-VASc and HAS-BLED data available for 59 of 60 patients.

### Traumatic events and TBI distribution

Over the study period, 43 patients (72%) sustained a single traumatic event, 12 (20%) sustained two events, four (6.7%) sustained three events, and one patient (1.7%) sustained four or more events. A total of 43 patients (72%) experienced at least one TBI; of these, 7 sustained two TBI events, yielding 50 TBI events available for analysis. Seventeen patients (28%) sustained traumatic events without TBI throughout the follow-up period (Table 1, Figure 1).

The first traumatic event occurred at a median of 12.0 years [IQR 7.5–18.0] after the index valve replacement operation, noting that this interval was approximated from year-level data as described in the Statistical Analysis section. The median patient age at the time of the first TBI was 73 years [IQR 56.5–79.8]. The predominant mechanism of injury was a ground-level fall, reported in 41 patients, while epileptic falls accounted for eight events. Among the remaining ground-level falls, the retrospective design precluded reliable systematic identification of neurological precipitants, including stroke or transient ischaemic attack as a consequence of valve thromboembolism, for individual fall events.

### TBI severity and intracranial hemorrhage

Of the 50 TBI events, 45 (90%) were classified as mild, four (8%) as moderate, and one (2%) as severe (Table 2). Intracranial hemorrhage was present in 30 of 50 TBI events (60%). The median hospital stay across all TBI events was 6 days [IQR 2–12], with data missing for two events.

**Table 2:** TBI characteristics and One-month outcomes.

Variable	All TBI Events (*n* = 50)
TBI severity, *n* (%)	
Mild	45 (90%)
Moderate	4 (8%)
Severe	1 (2%)
Intracranial hemorrhage, *n* (%)	30 (60%)
Surgical management (of hemorrhagic TBI), *n* (%)	9 (30%)
Hospital stay, days, median (IQR)^a^	6 (2–12)
Composite good outcome at one month, *n* (%)	
Overall	38/50 (76%)
Hemorrhagic TBI — surgical management	5/9 (55.6%)
Hemorrhagic TBI — conservative management	15/21 (71.4%) [*p* = 0.43 vs. surgical]
Non-hemorrhagic TBI — conservative management	18/20 (90.0%) [*p* = 0.09 vs. haemorrhagic]
Haemorrhage subtype — all haemorrhagic TBI (*n* = 30), *n* (%)	
Subdural haematoma	14 (46.7%)
Surgical	4 (44.4%)
Conservative	10 (47.6%)
Subarachnoid haemorrhage	13 (43.3%)
Surgical	4 (44.4%)
Conservative	9 (42.9%)
Intraparenchymal haemorrhage	3 (10.0%)
Surgical	1 (11.1%)
Conservative	2 (9.5%)
Epidural haematoma	0 (0%)
Admission INR, events with data available, *n* (%)	47/50 (94%)
Admission INR — all TBI events, median (IQR)	2.2 (1.6–3.1)
Admission INR — haemorrhagic TBI, median (IQR)	1.8 (1.4–2.6)
Admission INR — non-haemorrhagic TBI, median (IQR)	3.0 (1.9–3.3)*
Admission INR — haemorrhagic, surgical, median (IQR)	1.8 (1.2–2.5)
Admission INR — haemorrhagic, conservative, median (IQR)	2.0 (1.6–2.6)

a Hospital stay: two values missing. GCS cutoff definitions: mild 13–15, moderate 9–12, severe 8 or less. *p*-values from Fisher's exact test or Mann–Whitney U test as appropriate. Haemorrhage subtypes classified by primary type on admission CT. .

*p = 0.025 vs. haemorrhagic TBI (Mann–Whitney U). IQR, interquartile range; TBI, traumatic brain injury.

During hospitalisation, anticoagulation reversal or correction was administered in a subset of patients: 12 received phytomenadione (Konakion), four received four-factor prothrombin complex concentrate (Beriplex), two received tranexamic acid, and one received fresh frozen plasma (FFP). Additionally, low-molecular-weight heparin (LMWH) was documented in 27 TBI events, reflecting anticoagulation bridging or resumption rather than acute reversal. Anticoagulation reversal agent use stratified by haemorrhage status and composite good outcome is presented in Table 3.

**Table 3:** Anticoagulation management by haemorrhage Status and composite outcome (per TBI event).

Agent	Haemorrhagic TBI, *n*	Non-haemorrhagic TBI, *n*	Good outcome, *n*	Poor outcome, *n*
Phytomenadione (Konakion)	11	3	9	5
4F-PCC (Beriplex)	4	1	2	3
Tranexamic acid	2	0	0	2
Fresh frozen plasma (FFP)	1	0	1	0
LMWH (bridging/resumption)^a^	24	3	18	9
No anticoagulation intervention	3	16	18	1

a LMWH, low-molecular-weight heparin, used as anticoagulation bridging or resumption, not acute reversal. Events may appear in multiple rows if multiple agents were given. Formal statistical comparison not performed given small subgroup sizes. 4F-PCC, four-factor prothrombin complex concentrate.

### Management and outcomes of hemorrhagic TBI

Among the 30 TBI events complicated by intracranial haemorrhage, nine (30%) were managed surgically and 21 (70%) were managed conservatively. Haemorrhage subtype, classified by primary type on admission CT, was recorded for all 30 haemorrhagic TBI events: subdural haematoma was the primary haemorrhage type in 14 events (46.7%), subarachnoid haemorrhage in 13 events (43.3%), and intraparenchymal haemorrhage in 3 events (10.0%); no epidural haematoma was identified as the primary haemorrhage type. The distribution of haemorrhage subtypes was broadly similar between surgically and conservatively managed events (Table 2). Haemorrhage volume was not quantified and laterality was not systematically recorded. A composite good outcome at one month was achieved in 5 of 9 surgically managed haemorrhagic TBI events (55.6%) and in 15 of 21 conservatively managed haemorrhagic TBI events (71.4%) (*p* = 0.43). This comparison must be interpreted with extreme caution given the small number of surgically managed cases and the study's absence of statistical power (see Discussion). Among the 20 TBI events without intracranial haemorrhage, all were managed conservatively, and 18 of 20 (90.0%) achieved a composite good outcome (*p* = 0.09 vs. haemorrhagic TBI; Table 2, Figure 1).

Among patients with hemorrhagic TBI managed surgically, one patient died prior to the one-month follow-up, and one required reoperation. Among those managed conservatively with intracranial hemorrhage, three patients died prior to the one-month follow-up, and one patient had documented new hemorrhage at one month. Among the 20 TBI events without intracranial hemorrhage, one patient died prior to the one-month follow-up, and one patient — who had sustained a concomitant polytrauma at the time of the TBI — required reoperation for hip revision.

With respect to thromboembolic safety, four thromboembolic events were identified during TBI-related hospitalisations: one episode of mechanical valve thrombosis (in a patient with haemorrhagic TBI managed surgically, attributed to anticoagulation interruption, with poor composite outcome); one ischaemic stroke (in a patient with haemorrhagic TBI managed surgically, with good composite outcome); and two systemic thromboembolic events (one in a patient with haemorrhagic TBI managed conservatively, and one in a patient with non-haemorrhagic TBI managed conservatively, both achieving good composite outcome). No thromboembolic events were documented at the 30-day follow-up.

Admission INR data were retrievable for 47 of 50 TBI events (94%). The three events without available admission INR corresponded to pre-electronic-records-era TBI events (occurring in 1988 and 1996) for which no electronic laboratory record exists. The median admission INR across all events with available data was 2.2 [IQR 1.6–3.1]. Among haemorrhagic TBI events, the median admission INR was 1.8 [IQR 1.4–2.6], compared with 3.0 [IQR 1.9–3.3] among non-haemorrhagic TBI events (Mann–Whitney U, *p* = 0.025). Supratherapeutic INR (greater than 3.0) was present in 13 of 47 events (27.7%), of which 4 were haemorrhagic and 9 were non-haemorrhagic. Among haemorrhagic events, the median admission INR did not differ significantly between surgically managed events (1.8 [IQR 1.2–2.5]) and conservatively managed events (2.0 [IQR 1.6–2.6]; *p* = 0.423).

Overall, a composite good outcome was achieved in 38 of 50 TBI events (76%), with outcome rates differing across management groups and hemorrhage status, as summarized in Table 2.

## Discussion

The present study characterises the incidence, management, and one-month outcomes of traumatic brain injury in a single-centre cohort of patients with mechanical heart valves on lifelong anticoagulation, a population that has remained substantially underrepresented in the existing literature despite its unique and compounding clinical vulnerabilities (24). Our findings demonstrate that TBI is a frequent and clinically significant event in this population, occurring in nearly three quarters of patients over the study period, with intracranial haemorrhage complicating the majority of TBI events and conferring a measurably lower rate of composite good outcome compared to TBI without haemorrhage. The comparison between surgical and conservative management of haemorrhagic TBI must be regarded as strictly hypothesis-generating, given the study's absence of statistical power and the probable severity selection into the surgical group, as detailed below.

The high incidence of TBI observed in our cohort is consistent with the known hemorrhagic risk profile of vitamin K antagonist therapy and the epidemiology of fall-related injury in an aging anticoagulated population (6, 7). Of note, however, the median age at valve implantation in our cohort was 58 years, substantially younger than the populations described in the broader anticoagulated TBI literature, which has been driven predominantly by elderly patients anticoagulated for atrial fibrillation or venous thromboembolism (6, 12). The median age at first TBI of 73 years, occurring a median of 12 years after valve implantation, underscores the prolonged interval over which these patients accumulate traumatic risk — a temporal dimension that is unique to mechanical valve recipients and that distinguishes them from populations in whom anticoagulation is initiated in later life (1–3). This observation carries direct implications for long-term counseling and fall prevention strategies in younger patients undergoing mechanical valve replacement, a dimension of postoperative care that warrants greater clinical attention than it has hitherto received (8, 15).

The predominance of mild TBI in our cohort — accounting for 90% of events — is consistent with the epidemiology of ground-level falls as the primary injury mechanism, which was the case in the substantial majority of our patients (6, 7). Despite the mild severity classification of most events, intracranial hemorrhage was present in 60% of all TBI events, a rate that reflects the amplifying effect of therapeutic anticoagulation on hemorrhagic transformation following even low-energy cranial trauma (6, 7, 9, 24). This finding is concordant with published meta-analytic data demonstrating significantly elevated rates of intracranial hemorrhage in anticoagulated patients with mild TBI compared to non-anticoagulated counterparts (6, 12), and it reinforces the necessity of routine CT imaging in this population regardless of initial clinical severity, including consideration of repeat imaging to detect delayed hemorrhage (10, 11, 25). The high proportion of epileptic falls as an injury mechanism — accounting for eight events in our cohort — further highlights the neurological complexity of this population, in whom seizure activity may itself be both a consequence of prior intracranial events and a precipitant of further injury, a cycle that remains incompletely addressed in current management guidelines (15).

While direct oral anticoagulants (DOACs) remain categorically contraindicated in patients with mechanical prosthetic heart valves following the early termination of the RE-ALIGN trial due to excess valve thrombosis and stroke with dabigatran (21), comparative data from the broader anticoagulated TBI literature are informative. DOAC-treated patients have demonstrated lower haemorrhage expansion rates and more favourable neurological outcomes than VKA-treated counterparts following traumatic intracranial haemorrhage (7, 20, 22), highlighting the haemorrhagic vulnerability that VKA-dependent mechanical valve patients face and that cannot presently be mitigated by anticoagulant class switching. These findings further underscore the importance of research directed at anticoagulation-sparing strategies, including transcatheter valve alternatives in eligible patients, novel reduced-intensity antithrombotic regimens for specific valve designs, and development of biologically compatible prosthetic valve materials, as a long-term priority for reducing the haemorrhagic burden borne by mechanical valve recipients.

The management of intracranial haemorrhage in anticoagulated mechanical valve patients demands simultaneous control of active haemorrhage and prevention of valve thrombosis, competing imperatives that informed the reversal strategies observed in our cohort. The heterogeneity of reversal approaches reflects the absence of a universally accepted protocol and is consistent with the broader literature documenting significant practice variation (2, 4, 14, 15). The optimal timing of anticoagulation resumption after achieving haemorrhagic stability remains one of the most contested questions in this field; emerging evidence suggests that resumption within approximately 11 days may reduce the risk of recurrent haemorrhage while mitigating thromboembolic risk (16–18). Anticoagulation resumption timing was included in our data extraction protocol and retrieval was actively pursued; however, documentation of resumption decisions was systematically incomplete across the study period, reflecting fragmented inter-service communication and delegation of resumption decisions to outpatient physicians whose records were not consistently integrated into the institutional electronic health record. Reliable resumption timing data could not be recovered for a sufficient proportion of haemorrhagic TBI events to permit meaningful analysis, a major limitation that future prospective registry-based studies must specifically address.

The comparison between surgical and conservative management of haemorrhagic TBI in our cohort revealed composite good outcome rates of 55.6% and 71.4%, respectively (*p* = 0.43). This comparison must be interpreted with extreme caution: it involves only nine surgically managed cases, was not statistically significant, and the study was not designed or powered to detect a clinically meaningful difference. *post-hoc* power calculations indicate that approximately 120–150 events per group would be required to detect the observed 15.8% absolute difference with 80% power, an order of magnitude beyond the present cohort. Furthermore, surgical candidates almost certainly harboured more severe haemorrhages by virtue of the clinical indication for intervention, rendering the groups fundamentally non-comparable without formal haemorrhage severity adjustment; the absence of haemorrhage volume data precludes this adjustment. These findings are strictly hypothesis-generating, consistent with emerging data supporting conservative management for the majority of anticoagulation-associated traumatic intracranial haemorrhages (4, 14, 19). The difference in outcome rates between non-haemorrhagic TBI (90.0%) and haemorrhagic TBI (66.7%) (*p* = 0.09) reinforces intracranial haemorrhage as the primary determinant of unfavourable outcome (9, 11). The severity distribution, with only five moderate or severe events, precluded meaningful stratified analysis; prospective multicentre data are required.

Notably, admission INR was significantly higher in non-haemorrhagic TBI events than in haemorrhagic events (median 3.0 [IQR 1.9–3.3] vs. 1.8 [IQR 1.4–2.6]; *p* = 0.025), a finding that is counterintuitive but clinically informative: it suggests that biomechanical injury force, rather than the intensity of anticoagulation at the time of trauma, is the primary determinant of haemorrhagic transformation following traumatic brain injury in this population. Supratherapeutic anticoagulation alone was insufficient to predict intracranial haemorrhage occurrence in this cohort. These findings are hypothesis-generating and require prospective validation.

Several limitations of the present study merit explicit acknowledgement. First, the retrospective single-centre design introduces inherent selection bias and limits generalisability. Second, the modest cohort size constrains statistical power and precludes multivariable adjustment for confounders. Third, the comparison between surgical and conservative management of haemorrhagic TBI was never adequately powered; with only nine surgically managed cases, the observed *p*-value of 0.43 reflects the near-complete absence of statistical power, and probable severity selection into the surgical group renders the groups non-comparable without formal adjustment. This comparison must be regarded as strictly hypothesis-generating. Fourth, haemorrhage volume and laterality were not systematically recorded, precluding formal haemorrhage severity characterisation. Fifth, admission INR was unavailable for 3 of 50 TBI events (6%), corresponding to pre-electronic-records-era events for which no electronic patient data laboratory entries exist; no proxy INR was retrievable for these events. Sixth, anticoagulation resumption timing was actively sought but could not be systematically recovered owing to fragmented multi-clinic documentation across the 11-year study period. Seventh, the composite good outcome endpoint was assessed retrospectively without a validated neurological scale, and outcome assessors were not blinded to management strategy. Eighth, hospital-based retrospective ascertainment systematically excludes falls not resulting in emergency presentation, meaning true fall incidence almost certainly exceeds the rates reported here. Notwithstanding these limitations, this study provides a focused characterisation of a population consistently excluded from larger anticoagulation and TBI cohorts.

The findings of the present study carry several clinical implications. First, the high rate of intracranial hemorrhage following TBI — even in the setting of predominantly mild injuries — underscores the need for a low threshold for CT imaging and close in-hospital observation in all mechanical valve patients presenting after head trauma, irrespective of initial neurological status. Second, the favorable outcomes observed with conservative management in the majority of hemorrhagic TBI cases support a non-operative first approach in hemodynamically stable patients without clinical deterioration, consistent with emerging consensus in the broader anticoagulated TBI literature (14, 15, 23). Third, the prolonged interval between valve implantation and first TBI — with patients sustaining injuries a median of 12 years postoperatively at a median age of 73 years — highlights the importance of integrating fall risk assessment and prevention into the long-term follow-up of mechanical valve recipients from the time of implantation, a practice that is not consistently embedded in current cardiac surgical follow-up pathways. Prospective multicenter studies with standardized outcome definitions, systematic anticoagulation management protocols, and longer follow-up are needed to establish definitive evidence-based guidance for this population.

## Data Availability

The data analyzed in this study is subject to the following licenses/restrictions: Country restrictions on patient data. Requests to access these datasets should be directed to hector.rodriguez@usz.ch.

## References

[1] Wijdicks EFM Schievink WI Brown RD Mullany CJ. The dilemma of discontinuation of anticoagulation therapy for patients with intracranial hemorrhage and mechanical heart valves. Neurosurgery. (1998) 42(4):769–73. 10.1097/00006123-199804000-00053 9574641

[2] Kuramatsu JB Sembill JA Gerner ST Sprügel MI Hagen M Roeder SS. Management of therapeutic anticoagulation in patients with intracerebral haemorrhage and mechanical heart valves. Eur Heart J. (2018) 39(19):1709–23. 10.1093/eurheartj/ehy056 29529259 PMC5950928

[3] Lehto J Bjorn R Halminen O Linna M Haukka J Putaala J. Quality of anticoagulation and outcomes after mechanical aortic valve replacement in patients with atrial fibrillation: a nationwide cohort study. Eur Heart J. (2023) 44(Supplement_2). 10.1093/eurheartj/ehad655.1640 PMC12342859 40366898

[4] Bashline M Chaudhary R Wong T Okonkwo DO Aiyer A. Examining the duration and safety of withholding anticoagulation after an intracerebral hemorrhage in patients with mechanical heart valves. Am J Cardiol. (2022) 177:164–6. 10.1016/j.amjcard.2022.05.008 35764429

[5] Romualdi E Micieli E Ageno W Squizzato A. Oral anticoagulant therapy in patients with mechanical heart valve and intracranial haemorrhage. Thromb Haemost. (2009) 101(2):290–7. 10.1160/TH08-07-0474 19190812

[6] Karamian A Seifi A Karamian A Lucke-Wold B. Incidence of intracranial bleeding in mild traumatic brain injury patients taking oral anticoagulants: a systematic review and meta-analysis. J Neurol. (2024) 271:3849–68. 10.1007/s00415-024-12424-y 38755424

[7] Cipriano A Park N Pecori A Bionda A Bardini M Frassi F. Predictors of post-traumatic complication of mild brain injury in anticoagulated patients: dOACs are safer than VKAs. Intern Emerg Med. (2021) 16:2143–52. 10.1007/s11739-020-02576-w 33386604

[8] Menditto VG Rossetti G Sampaolesi M Buzzo M Pomponio G. Traumatic brain injury in patients under anticoagulant therapy: review of management in emergency department. J Clin Med. (2024) 13(13):3669. 10.3390/jcm13133669 38999235 PMC11242576

[9] Botros D Gautam D Hamrick FA Nguyen S Cortez J Young JB. Impact of premorbid oral anticoagulant use on survival in patients with traumatic intracranial hemorrhage. Neurosurg Focus. (2023) 55(4):E7. 10.3171/2023.7.FOCUS23380 37778038

[10] Capsoni N Carpani G Tarantino F Gheda S Cugnod JM Lanfranchi S. Incidence and risk factors for delayed intracranial hemorrhage after mild brain injury in anticoagulated patients: a multicenter retrospective study. Scand J Trauma Resusc Emerg Med. (2025) 33:29. 10.1186/s13049-025-01337-y 39930444 PMC11808940

[11] Giamello JD Martini G Fulcheri C Visconti GL Curtetti S Lakehal J. Impact of anticoagulant therapy on delayed intracranial haemorrhage after traumatic brain injury: a study on the role of repeat CT scans and extended observation. Injury. (2025) 56(9):112523. 10.1016/j.injury.2025.112523 40537351

[12] Santing JL Lee YX van der Naalt J van den Brand CL Jellema K. Mild traumatic brain injury in elderly patients receiving direct oral anticoagulants: a systematic review and meta-analysis. J Neurotrauma. (2022) 39(7–8):488–500. 10.1089/neu.2021.0435 35057639

[13] Gao W Zhang Z Jia P Dong L Li R Xu J. Efficacy and safety of direct oral anticoagulants in patients after heart valve replacement or repair: a systematic review and network meta-analysis. Clin Ther. (2025) 47(11):1061–8. 10.1016/j.clinthera.2025.08.017 40993026

[14] Samuel S Alnosair E. Increased bleeding risk with bridging and early anticoagulation in traumatic brain injury patients with intracranial hemorrhage and mechanical heart valves. Neurosurg Rev. (2025) 48:215. 10.1007/s10143-025-03702-y 40624381

[15] Wiegele M Schöchl H Haushofer A Ortler M Leitgeb J Kwasny O. Diagnostic and therapeutic approach in adult patients with traumatic brain injury receiving oral anticoagulant therapy: an Austrian interdisciplinary consensus statement. Crit Care. (2019) 23(1):74. 10.1186/s13054-019-2352-6 30795779 PMC6387521

[16] Barra ME Forman R Long-Fazio B Merkler AE Gurol ME Izzy S. Optimal timing for resumption of anticoagulation after intracranial hemorrhage in patients with mechanical heart valves. J Am Heart Assoc. (2024) 13(3):e032094. 10.1161/JAHA.123.032094 38761076 PMC11179836

[17] Sakusic A Rabinstein AA Anisetti B Mandrekar J Wijdicks EFM Freeman WD. Timing of anticoagulation resumption and risk of ischemic and hemorrhagic complications in patients with ICH and mechanical heart valves. Neurology. (2024) 102(3):e209664. 10.1212/WNL.0000000000209664 39102615

[18] D'Anna L Prandin G Pirera E Sacco S Veltkamp R Korompoki E. Timing and safety of anticoagulation reinitiation after intracranial hemorrhage in patients with mechanical valves. Neurology. (2025) 105:e214184. 10.1212/WNL.0000000000214184 40997282 PMC12473174

[19] Khan S Syed F Toma M. Management of intracranial hemorrhage in the setting of mechanical heart valve replacement therapy. Appl Mech. (2023) 4(2):584–94. 10.3390/applmech4020033

[20] Antoni A Wedrich L Schauperl M Höchtl-Lee L Sigmund IK Gregori M. Management of traumatic brain injury in patients with DOAC therapy—are the “new”. Oral Anticoagulants Really Safer? J Clin Med. (2022) 11(21):6268. 10.3390/jcm11216268 36362496 PMC9658566

[21] Eikelboom JW Connolly SJ Granger CB Kappetein AP Mack MJ Blatchford J. Dabigatran versus warfarin in patients with mechanical heart valves. N Engl J Med. (2013) 369(13):1206–14. 10.1056/NEJMoa1300615 23991661

[22] Shin SS Marsh EB Ali H Nyquist PA Hanley DF Ziai WC. Comparison of traumatic intracranial hemorrhage expansion and outcomes among patients on direct oral anticoagulants versus vitamin K antagonists. Neurocrit Care. (2020) 32(2):407–18. 10.1007/s12028-019-00898-y 32034657

[23] King B Milling T Gajewski B Costantini TW Wick J Price MA. Restarting and timing of oral anticoagulation after traumatic intracranial hemorrhage: a review and summary of ongoing and planned prospective randomized clinical trials. Trauma Surg Acute Care Open. (2020) 5(1):e000605. 10.1136/tsaco-2020-000605 33313417 PMC7716676

[24] Lim XT Ang E Lee ZX Hajibandeh S Hajibandeh S. Prognostic significance of preinjury anticoagulation in patients with traumatic brain injury: systematic review and meta-analysis. J Trauma Acute Care Surg. (2021) 90(1):191–201. 10.1097/TA.0000000000002976 33048909

[25] Hadwe S Assamadi M Barrit S Giannis D Haidich AB Goulis DG. Delayed intracranial hemorrhage of patients with mild traumatic brain injury under antithrombotics on routine repeat CT scan: systematic review and meta-analysis. Brain Inj. (2022) 36(6):703–13. 10.1080/02699052.2022.2065034 35476710

[26] Gaetani P Revay M Sciacca S Pessina F Aimar E Levi D. Traumatic brain injury in the elderly: considerations in a series of 103 patients older than 70. J Neurosurg Sci. (2012) 56(3):231–7. 22854591

